# LINC00467 facilitates the proliferation, migration and invasion of glioma via promoting the expression of inositol hexakisphosphate kinase 2 by binding to miR-339-3p

**DOI:** 10.1080/21655979.2021.2018098

**Published:** 2022-02-13

**Authors:** Yin Zhang, Yaxuan Zhang, Sen Wang, Boqiang Cao, Daling Hu, Junli Jia, Yuhang Wang, Luyao Chen, Jiaming Li, Huamin Tang, Hongyi Liu

**Affiliations:** a Department of Neurosurgery, Sir Run Run Hospital, Nanjing Medical University Nanjing, China; b School of Basic Medical Sciences, Nanjing Medical University, Nanjing China; c Department of Geriatrics, Sir Run Run Hospital, Nanjing Medical University, Nanjing, China; d Department of Neurosurgery, The Affiliated Brain Hospital of Nanjing Medical University, Nanjing, China

**Keywords:** Glioma, LINC00467, miR-339-3p, IP6K2, malignant progression

## Abstract

Our previous studies indicate that long noncoding RNA (lncRNA) LINC00467 can act as an oncogene to participate in the malignant progression of glioma, but the underlying molecular mechanism remains to be studied further. This study aimed to explore the biological role of the LINC00467/miR-339-3p/ inositol hexakisphosphate kinase 2 (IP6K2) regulatory axis in glioma. The Cancer Genome Atlas (TCGA), Oncomine databases and reverse transcription‑quantitative PCR (RT‑qPCR) were used to analyze IP6K2 expression in glioma. RT-PCR, EdU and transwell assays were conducted to observe the effect of IP6K2 on glioma cell proliferation, migration and invasion. Using bioinformatics analysis, RT-PCR, and dual luciferase reporter gene assay, the potential role of the LINC00467/miR-339-3p/IP6K2 regulatory axis in glioma was verified. The results showed that IP6K2 was up-regulated in glioma tissues and cell lines. Moreover, the expression level of IP6K2 was correlated with the clinical features of glioma patients. *In vitro* and *in vivo* experiments indicated that IP6K2 overexpression could promote the proliferation, migration, and invasion of glioma cells. Further bioinformatics analysis and *in vitro* assays revealed that LINC00467 could promote IP6K2 expression by binding to miR-339-3p and promote the malignant progression of glioma. Overall, LINC00467 could upregulate IP6K2 by binding to miR-339-3p and promote the proliferation, migration, and invasion of glioma cells. The LINC00467/miR-339-3p/IP6K2 regulatory axis might be a potential therapeutic target for glioma.

## Introduction

Glioma is one of the most common and aggressive malignant tumors of the nervous system, accounting for approximately 30% of central nervous system (CNS) tumors and 80% of primary malignant brain tumors, with characteristics of highly invasive growth [1,2]. In its early stages, glioma does not show obvious clinical symptoms because the cancer is small and located in the skull; therefore, it is often difficult to detect until it grows to a size large enough to exert intracranial compression and other central nervous system-related symptoms [3,4]. Although the treatment approaches for glioma, such as surgical treatment combined with radiotherapy, drug chemotherapy, and other treatment methods, have advanced greatly in recent years, the prognosis of patients with advanced glioma remains poor [5,6]. Therefore, there is an urgent need to explore novel targets for early diagnosis and specific intervention in glioma.

Long noncoding RNAs (lncRNAs) are a class of non-coding RNA molecules with a length of more than 200 nucleotides. Owing to the lack of continuous open reading frames (ORFs), most lncRNAs lack evident protein-coding ability. LncRNAs are widely involved in biological processes including gene imprinting, RNA splicing, and chromosome modification [7,8]. Current research indicates that lncRNAs play a crucial role in various cell functions by regulating chromosome structure, RNA transcription, editing, transportation, and organelle production [9,10]. Many lncRNAs have been found to control tumor growth and differentiation by directly acting on coding genes [11,12]. Further, lncRNA expression shows strong tissue specificity, and abnormal expression of lncRNA may serve as a biomarker for assessing the progress and prognosis of glioma [13,14].

With increasingly detailed research on microRNAs (miRNAs) in the past decades, the roles and mechanisms of miRNAs in tumors are relatively well known. miRNAs are small non-coding RNAs with a length of 20–25 nucleotides, and they inhibit the expression of target genes by specifically binding to the messenger RNAs (mRNAs) sequence [15,16]. As an essential member of the ncRNA family, lncRNAs have been reported to exert their biological functions in malignant tumors by interacting with miRNAs and are thus known to participate in tumor occurrence and development. Salmena et al[17], proposed a competitive endogenous RNA (ceRNA) hypothesis stating that mRNAs, transcription pseudogenes, and lncRNAs are natural miRNA sponges. Ballantyne et al [18]. also reported that lncRNAs could regulate miRNA function by acting as an endogenous sponge, thereby controlling the expression of target genes. in a previous study, we found increased LINC00467 expression in glioma tissues, and that LINC00467 could inhibit p53 expression in the nucleus by binding to DNA methyltransferase 1 (DNMT1), and facilitate glioma cell proliferation, migration, and invasion [19]. However, whether LINC00467 can modulate the expression of other target genes by sponging miRNAs in the cytoplasm remains unknown.

In this study, we found that IP6K2 expression is significantly increased in gliomas upon analyzing bioinformatics databases, but its biological effects and potential molecular mechanisms remain unclear. Through bioinformatic analysis and cell-based experiments, we found that IP6K2 and LINC00467 might simultaneously bind to miR-339-3p. Therefore, this study aimed to explore the possible biological effects and therapeutic potential of LINC00467/miR-339-3p/IP6K2 in glioma.

## Materials and methods

### Bioinformatics analysis

The expression level of IP6K2 in tumors was analyzed using the TIMER 2.0 database (http://timer.comp-genomics.org/) [20]. GEPIA (http://gepiacancer-pku.cn/index.html) was used to analyze the expression level of IP6K2 in 163 glioma tissues and 207 normal control tissues; the overall survival (OS) and disease-free survival (DFS) rates of patients with were computed using the GEPIA database [21]. The Oncomine database (http://www.oncomine.org) was used to analyze the expression levels in 81 glioma tissue samples and 23 normal control tissue samples [22]. The miRNAs that could simultaneously bind to LINC00467 and IP6K2 were predicted using the Starbase database (http://starbase.sysu.edu.cn/) [23].

### Sample collection

30 glioma tissue samples and 30 normal brain tissues from patients who were non-glioma diseases were collected from Sir Run Run Hospital, Nanjing Medical University and the Affiliated Brain Hospital of Nanjing Medical University. All tissues were confirmed by pathological examination. This study was approved by the hospital ethics committee. All patients voluntarily participated in the study and provided written informed consent. All experiments were conducted in accordance with the Declaration of Helsinki. The sample tissues were quickly frozen with liquid nitrogen after isolation and were stored at −80°C until used. Detailed information is presented in Table 1.

**Table 1.** Clinical features of 30 glioma patients and the expression of IP6K2

Parameters	Group	Cases	IP6K2 expression		*P*-value
			High	Low	
Gender	Male	18	9	9	0.722
	Female	12	7	5	
Age at surgery	≥ 60	20	12	8	0.442
	< 60	10	4	6	
WHO stage	I+ II	13	3	10	0.009
	III+IV	17	13	4	
Tumor size (maximum diameter in MRI)	≥ 30 mm	13	7	6	0.713
	< 30 mm	17	7	10	
Distant metastasis	Yes	16	12	4	0.026
	No	14	4	10	

### Cell culture and transfection

The normal control cell line, HEB, and human glioma cell lines (LN229, LN308, U87, and U251) were purchased from the American Type Culture Collection (ATCC, Rockville, MD, USA). All the above cells were cultured in Dulbecco’s modified Eagle medium (DMEM, Gibco, USA) containing 10% fetal bovine serum (FBS, Invitrogen, USA) and placed in an environment at 37°C with 5% CO_2_. Following the manufacturer’s instructions for Lipofectamine 3000 (Invitrogen, USA), we used IP6K2 short hairpin RNAs (shRNAs) and overexpression plasmid, miR-339-3p mimics and inhibitor, and the corresponding negative control for cell transfection. All transfection reagents were designed by GenePharma (Shanghai, China). To establish the LV-IP6K2 stable expression cell line, we first inoculated the cells to be transfected (U87) into a 6-well plate at a density of 2 × 10^5^ cells/ml, with 2 ml of cell suspension per well, and cultured them overnight. We then replaced the medium with a medium containing lentivirus (1 ml 10% DMEM with 10% fetal bovine serum + polybrene (final concentration 8 μg/ml) + 1 ml virus), and mixed well. After 12 h, the medium was replaced with fresh DMEM containing 10% fetal calf serum. After 48 h, the medium was aspirated and replaced with a medium containing the lowest concentration of puromycin for selecting stably expressing cells.

### RNA extraction and reverse transcription‑quantitative PCR (RT‑qPCR)

TRIzol reagent (Invitrogen, USA) was used to extract RNA from glioma tissues and cells. Complementary DNA (cDNA) was prepared by reverse transcription using the PrimeScript RT Reagent Kit (TAKARA, Code No. RR036A), according to the manufacturer’s instructions [24]. The RT-PCR assay was performed using the SYBR® Green Master Mix (TaKaRa) on the ABI 7500 system. GAPDH and U6 were used as internal controls, and the relative gene expression was calculated as 2^−ΔΔCT^ [25].The primer sequences used were as follows: LINC00467: Forward: 5ʹ- GCCAGAGCAAGACTCTGTCTAC-3ʹ, Reverse: 5ʹ- GATGGGATACACATTCAATCAT-3ʹ miR-339-3p: Forward, 5ʹ-CCGCTCTCCCTGTCCTCC-3ʹ, Reverse: 5ʹ-CAGACACTGGGGCAGGC-3ʹ IP6K2: Forward: 5ʹ-TCCCCACCCTGGTATAGTCC-3ʹ, Reverse: 5ʹ-CCATCTCAAACCCTGGACCC-3ʹ GAPDH: Forward: 5ʹ‐CGGAGTCAACGGATTTGGTCGTAT‐3ʹ, Reverse: 5ʹ‐AGCCTTCTCCATGGTGGTGAAGAC‐3ʹ and U6: Forward: 5ʹ-GCTGAGGTGACGGTCTCAAA‐3ʹ, reverse: 5ʹ-GCCTCCCAGTTTCATGGACA‐3ʹ.

### 5-ethynyl-2ʹ-deoxyuridine (EdU) assay

The EdU kit (RiboBio, China) was used to examine cell proliferative ability according to the previous study [26]. Cells were seeded into a 96-well plate at a density of 3000 cells/well, and 50 mM EdU solution was added to the medium. After 24 h, the cells were fixed with 4% formaldehyde and permeabilized with Triton X-100. The treated cells were then incubated with the EdU reaction mixture and counterstained with Hoechst. Staining results were recorded using a fluorescence microscope. Five microscopic fields of view were selected at random to count the number of EdU bound cells.

### Transwell assay

We conducted the transwell assay according to the previous study [27]. Brifely, U87 and U251 cells were inoculated into the upper chamber of the Transwell insert in 100 μL of serum-free DMEM at a density of 5 × 10^4^ cells/well (invasion experiments require 100 μg of matrix gel to be pre-coated in the upper chamber of the cell), whereas the lower chamber was filled with 600 μl of completed culture medium. After culturing in an incubator at 37°C with 5% CO_2_ for 24 h, the cells in the upper chamber were wiped with a cotton swab, and the cells migrated to the lower part of the Transwell membrane were fixed with methanol for 25 min, and stained with crystal violet for 30 min. Five fields of view were randomly selected for microscopic imaging (X200) and the cells were then counted.

### Dual luciferase reporter gene experiment

We conducted the dual-luciferase reporter gene experiment according to the previous study [28]. Brifely, bioinformatics websites were analyzed to predict the binding sites of miR-339-3p, LINC00467, or IP6K2. Next, 5 × 10^4^ U87 and U251 cells were seeded in a 24-well plate. After 48 h of co-transfection with the corresponding plasmid and miR-339-3p mimics or negative control, a Promega kit (Promega, Madison, WI, USA) was used to measure and record the luciferase activity according to the manufacturer’s instructions. The experiment was repeated three times independently, and the mean value was used for statistical analysis.

### Western blot assay

We conducted the Western blot assay according to the previous study [29]. Brifely, treated glioma cells were collected and lysed using a lysis buffer containing the protease inhibitor PMSF (Beyotime, Nantong, China), according to the BCA protein quantification kit (Beyotime, Nantong, China) to measure the protein concentrations. SDS-PAGE protein loading buffer was added, and the mixture was heated at 100°C to denature the protein. After loading and running, the separated proteins were transferred to a PVDF membrane, which was then cut according to the molecular weight. Antigen blocking was performed with 5% skimmed milk powder blocking solution. Antibodies against IP6K2 (ab179921; 1/1000) and GAPDH (ab8245; 1/1000) were purchased from Abcam (Cambridge, MA, USA). HRP-conjugated secondary goat anti-mouse and goat anti-rabbit antibodies (Proteintech, USA) were then added to the membrane and incubated. The membranes were then immersed in ECL Plus (Millipore, USA) at room temperature. Protein expression levels were detected using a Bio-Imaging System (Bio-Rad, USA).

### Xenograft mouse models

We chose 4-week-old BALB/c-nude mice for the in vivo experiments. Cell lines stably overexpressing IP6K2 in the logarithmic growth phase and the control cell lines were dissociated with trypsin and adjusted to 5 × 10^7^ cells/ml in 100 μl medium. A syringe was used to inject the cells subcutaneously into the right abdomen of nude mice. Tumor growth was measured every week, and recorded the length (L) and short diameter (W) of the tumor, were measured to calculate the volume as V = LW [2,2^30^], and drew the tumor growth curve. After 5 weeks, the mice were euthanized and photographed according to the regulations.

### Statistical Methods

Statistical analysis was performed using SPSS 22.0, and the measured data were expressed as the mean ± standard deviation; a two-sample t-test was used for comparison between groups. GraphPad Prism 6 software was used to perform the relevant statistical analyses and graphing. Each experiment was independently performed three times, and the results are expressed as means ± SD. Statistical significance was set at p < 0.05.

## Results

This study aimed to demonstrate the expression level, biological function and potential mechanism of IP6K2 in glioma. We hypothesized that IP6K2 could function as a vital role in the maglinant progression of glioma. Based on our previous study and results of assays, we demonstrated that LINC00467 was obviously up-regulated in glioma tissues and cell lines, and LINC00467 could sponge miR-339-3p to up-regulate IP6K2 expression in cytoplasm and promote glioma progression. Overall, this study verified the function and mechanism of the LINC00467/miR-339-3p/IP6K2 regulatory axis in glioma, which might provide a novel insight for glioma diagnosis and therapy.

### IP6K2 was upregulated in glioma tissues

**Figure 1.**
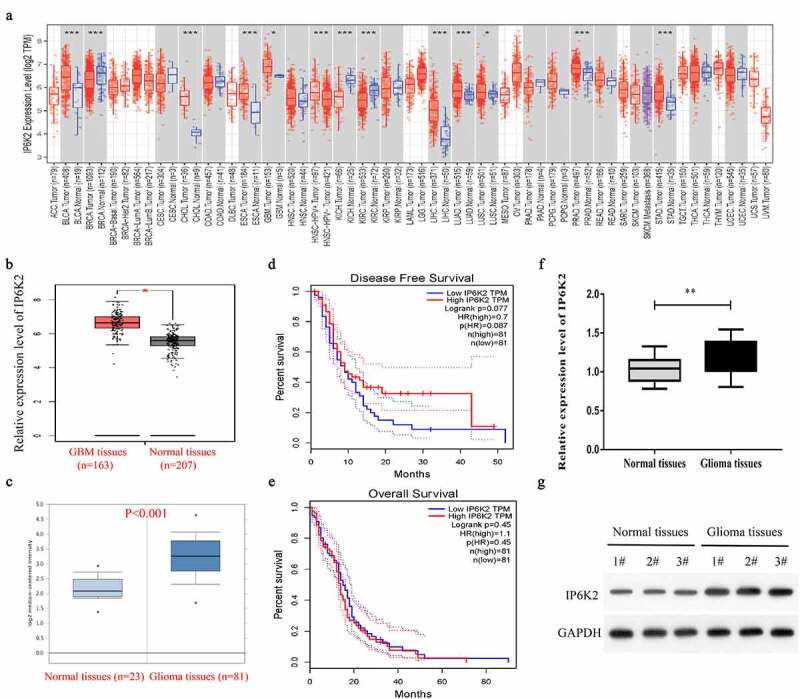
**IP6K2 exhibited high expression in glioma tissues** A Analyze the expression level of IP6K2 in a variety of tumors through the TIMER2.0 database; B Analyze the expression of IP6K2 in 163 glioma tissues and 207 normal tissues through the TCGA database; C Analyze the expression of IP6K2 in 23 glial tissues through the Oncomine database The expression of tumor tissue and 81 normal tissues; D Analyze the correlation between IP6K2 expression and the overall survival rate (OS) of glioma patients through TCGA database; E Analyze the relationship between IP6K2 expression level and disease-free survival of glioma patients through TCGA database (DFS) correlation; F Detected the mRNA expression of IP6K2 in glioma tissues and normal control tissues by RT-PCR assay; G Detected the protein expression of IP6K2 in glioma tissues and normal control tissues by Western blot assay. *p < 0.05; ** p < 0.01; *** p < 0.001.

We first analyzed the expression level of IP6K2 in tumors using the TIMER2.0 database. As shown in Figure 1(a), the expression levels of IP6K2 in bladder cancer (BLCA), cholangiocarcinoma (CHOL), esophageal carcinoma (ESCA), glioblastoma multiforme (GBM), liver hepatocellular carcinoma (LIHC), lung adenocarcinoma (LUAD), lung squamous cell carcinoma (LUSC), prostate adenocarcinoma (PRAD), and stomach adenocarcinoma (STAD) were significantly increased; however, in breast invasive carcinoma (BRCA), kidney chromophobe (KICH), and kidney renal clear cell carcinoma (KIRC), IP6K2 showed decreased expression. Further analysis of TCGA and Oncomine databases showed that IP6K2 expression in glioma tissues was significantly higher than that in adjacent tissues (Figure 1(b-c)). In addition, the expression level of IP6K2 was not significantly correlated with the overall survival (OS) and disease-free survival (DFS) rates in patients with glioma (Figure 1(d-e)). However, In our study, the Kaplan–Meier analysis indicated that glioma patients with high IP6K2 expression had a poor OS outcome (Figure S1A), but had no correlation with DFS (Figure S1B). We measured the expression of IP6K2 in 30 glioma tissue samples and 30 normal control tissues using RT-PCR. The results indicated that the expression of IP6K2 in glioma tissues was significantly higher (Figure 1(f)). Further, Western blot results confirmed that the protein expression level of IP6K2 in glioma tissues was also significantly increased (Figure 1(g)). Moreover, in our validation cohort, IP6K2 was elevated in patients with advanced stages of glioma (III + IV vs I + II, P = 0.009) and metastatic glioma (P = 0.026) (Table 1). These results indicate that IP6K2 might have an essential cancer-promoting effect in gliomas.

### IP6K2 overexpression could promote cells to proliferate, migrate, and invade

**Figure 2.**
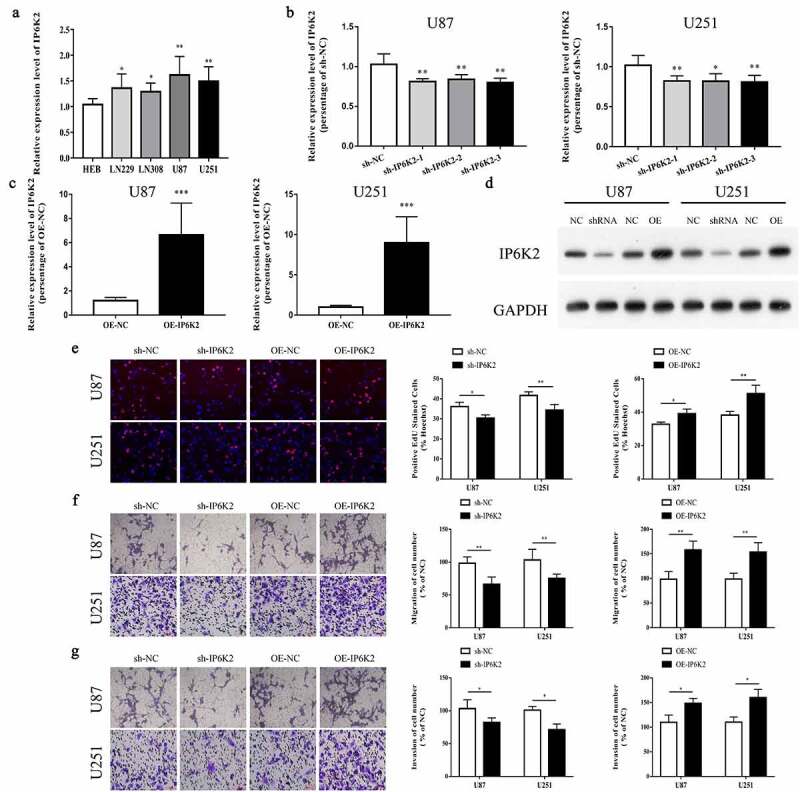
**Overexpression of IP6K2 could promote cells to proliferate, migrate and invade** A The mRNA expression of IP6K2 in glioma cells and normal control cells was detected by RT-PCR; B U87 and U251 were down-regulated by small interfering RNA (sh-IP6K2-1, sh-IP6K2-2 and sh-IP6K2-3) The expression of IP6K2 in the cells was verified by RT-PCR; C The expression of IP6K2 in U87 and U251 cells was up-regulated by overexpression plasmids and verified by RT-PCR; D Staining efficiency was verified by Western blot experiments for the conversion of sh-IP6K2-1 and OE-IP6K2; E The effect of IP6K2 on the proliferation of U87 and U251 cells was tested by EdU experiment. (Magnification: 200X) F-G Transwell experiment was conducted to detect changes in cell migration and invasion ability after inhibiting or overexpressing IP6K2 in U87 and U251 cell lines. (Magnification: 200X) *p < 0.05; ** p < 0.01.

IP6K2 in glioma cell lines was also remarkably higher than that in normal control cells (Figure 2(a)). To probe the biological effects of IP6K2 in gliomas, we used shRNAs or overexpression plasmids (OE) to inhibit or overexpress IP6K2 in U87 and U251 cells, and used RT-PCR to verify the transfection efficiency. The results showed that IP6K2 shRNAs significantly inhibited the expression of IP6K2, and that sh-IP6K2-1 showed a higher interference efficiency (Figure 2(b)). OE-IP6K2 significantly promoted the expression of IP6K2 in glioma cells (Figure 2(c)). In addition, Western blot experiments showed that si-IP6K2 and OE-IP6K2 significantly inhibited or increased the protein expression level of IP6K2 in U87 and U251 cells (Figure 2(d)). The EdU experiment verified that sh-IP6K2 significantly inhibited the proliferation of U87 and U251 cells, whereas OE-IP6K2 promoted cell growth (Figure 2(e)). Transwell experiments revealed that, compared with the sh-NC group, the migration and invasion ability of U87 and U251 cells in the sh-IP6K2 group was significantly weakened, whereas IP6K2 overexpression had the opposite effect (Figure 2(f-g)). The above results demonstrated that IP6K2 promoted the proliferation, migration, and invasion of glioma cells.

### IP6K2 overexpression promoted glioma tumor growth in vivo

**Figure 3.**
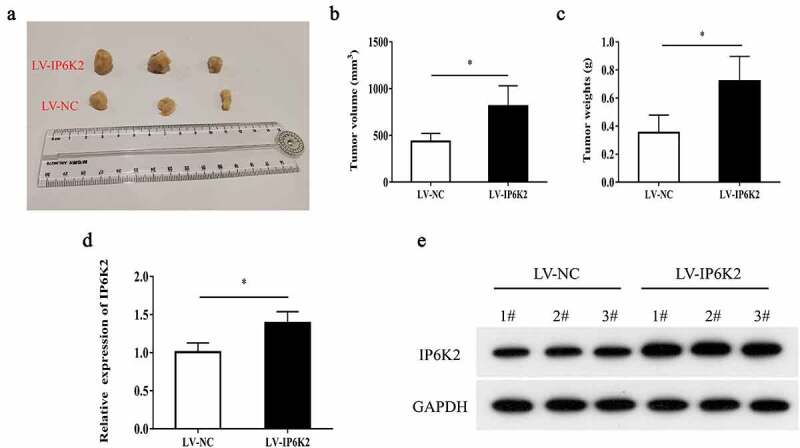
**Overexpression of IP6K2 promoted the growth of glioma in vivo** A Representative images of tumors in nude mice in each group; B Determine tumor volume; C Determine tumor weight; D RT-PCR showed that the expression of IP6K2 was increased in the tumor tissues of the LV-IP6K2 group; E Western blot showed that IP6K2 was expressed in the LV-IP6K2 group Increased in the tumor tissue. *p < 0.05.

To further confirm that IP6K2 is involved in glioma progression, stable lentivirus-mediated IP6K2 overexpressing U87 cells or LV-transfected U87 cells were used for tumor formation in immunodeficient mice. Compared with the LV-NC group in vivo, IP6K2 upregulation increased the tumor volume and weight (Figure 3(a-c)). According to RT-PCR and Western blot analysis, IP6K2 overexpression upregulated both IP6K2 mRNA and protein expression (Figure 3(d-e)). Further, IP6K2 overexpression significantly promoted glioma tumor growth in vivo, which is consistent with our in vitro results.

### MiR-339-3p could both bind to LINC00467 and IP6K2

**Figure 4.**
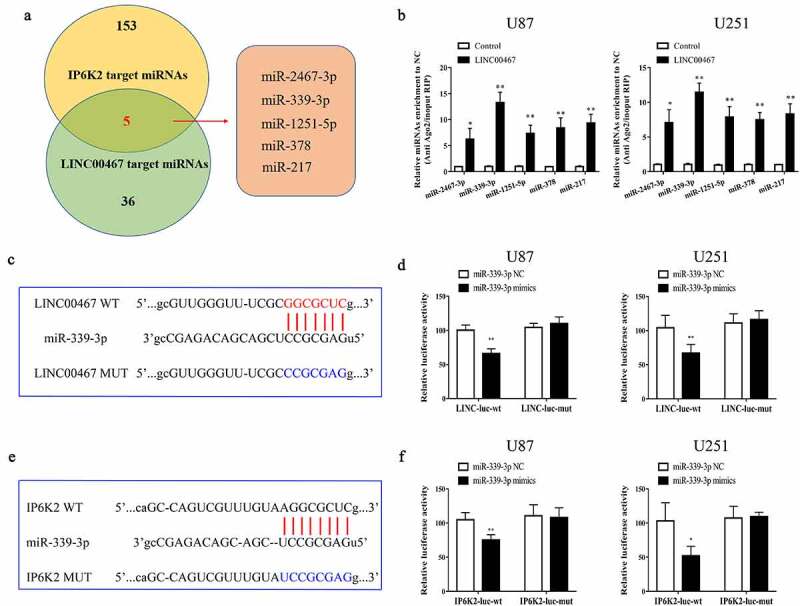
**MiR-339-3p could bind to LINC00467 and IP6K2 at the same time** A Bioinformatics predicts miRNAs that may bind to LINC00467 and IP6K2, including miR-2467-3p, miR-339-3p, miR-1251-5p, miR-378 and miR-217; B Discover miR on the Kean side of the RIP experiment −339-3p has the strongest binding ability with LINC00467; C Construct LINC00467 wild-type plasmid (LINC00467-WT) and LINC00467 mutant plasmid (LINC00467-MUT) according to the binding site; D It is found through the double luciferase reporter gene experiment test, miR-339-3p can be combined with LINC00467; E Construction of IP6K2 wild-type plasmid (IP6K2-WT) and IP6K2 mutant plasmid (IP6K2-MUT) based on the binding site; D Through the double luciferase reporter gene test, it was found that miR- 339-3p can bind to IP6K2. *p < 0.05; **p < 0.01.

Our previous study showed that LINC00467 in the nucleus could promote the malignant progression of glioma by regulating p53 expression in combination with DNMT1^19^; however, its mechanism in the cytoplasm is not yet studied. First, we detected the expression of LINC00467 in glioma tissues and found that the expression of LINC00467 in glioma tissues was significantly higher (Figure S2A). Moreover, the epression level of LINC00467 was positively correlated with IP6K2 expression (Figure S2B). Then, we investigated whether LINC00467 could regulate IP6K2 expression in the cytoplasm through the ceRNA mechanism. Bioinformatics analysis (http://starbase.sysu.edu.cn/) predicted miRNAs that could simultaneously bind LINC00467 and IP6K2. The results indicated five candidate miRNAs (miR-2467-3p, miR-339-3p, miR-1251-5p, miR-378, and miR-217) as potential targets (Figure 4(a)). Subsequently, the RIP experiment showed that miR-339-3p had the strongest binding ability with LINC00467 (Figure 4(b)). Therefore, we selected miR-339-3p for follow-up research. The LINC00467 wild-type plasmid (LINC00467 WT) and the LINC00467 mutant plasmid (LINC00467 MUT) (Figure 4(c)) were designed and constructed based on the predicted binding sites. Using the dual luciferase reporter gene assay, we found that miR-339-3p could bind to LINC00467 (Figure 4(d)). Similarly, we designed and constructed an IP6K2 wild-type plasmid (IP6K2 WT) and IP6K2 mutant plasmid (IP6K2 MUT) based on the predicted binding sites (Figure 4(e)). The results of the dual-reporter gene assay indicated that miR-339-3p could bind to IP6K2 (Figure 4(f)). The above results indicate that LINC00467 might participate in the occurrence and progression of glioma by competitively binding miR-339-3p along with IP6K2.

### The LINC00467/miR-339-3p/IP6K2 regulatory axis promotes the proliferation, migration, and invasion of glioma cells

**Figure 5.**
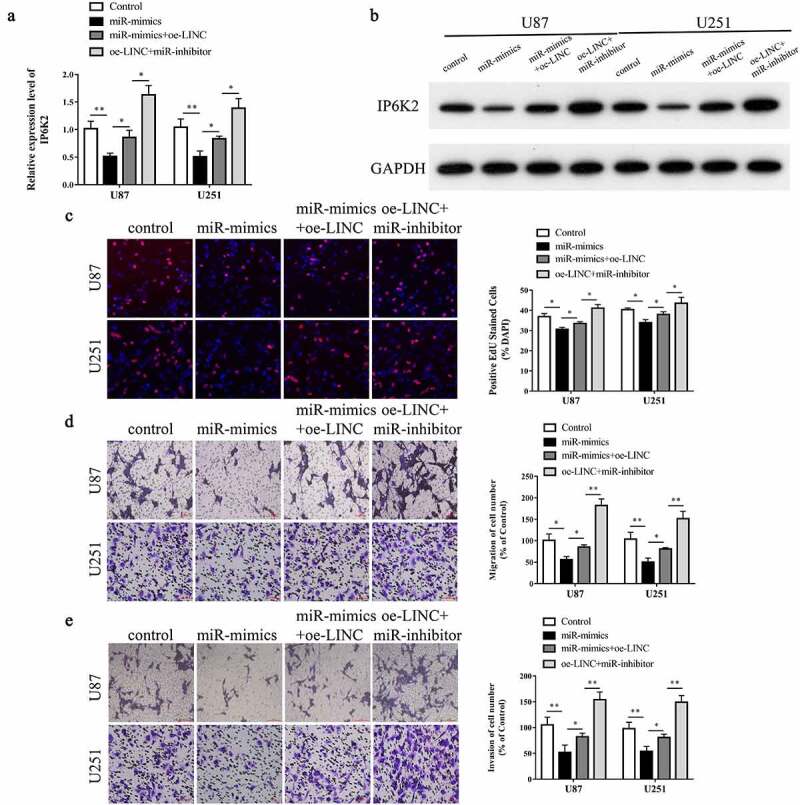
**LINC00467/miR-339-3p/IP6K2 regulatory axis promoted the proliferation, migration and invasion of glioma cells** A-B After U87 and U251 cells were simultaneously transfected with miR-339-3p mimics, miR-339-3p inhibitor and LINC00467 overexpression plasmids, the expression of IP6K2 was detected by RT-PCR and Western blot experiments; C The effect of LINC00467/miR −339-3p/IP6K2 regulation axis on the proliferation of glioma cells was detected by EdU experiment; D-E Transwell experiment was used to detect the effect of LINC00467/miR-339-3p/IP6K2 regulation axis on the migration and invasion of glioma cells. *p < 0.05; ** p < 0.01.

To further examined the potantial mechanism of LINC00467 in glioma, we detected the proliferative, migrative and invasive abilities of glioma cells after transfection of U87 and U251 cells with LINC00467 siRNA and miR-339-3p inhibitor. The results indicated showed that after inhibition of LINC00467 in U87 and U251 cells could significantly inhibit cell proliferation, migration, and invasion, whereas simultaneous downregulation of miR-339-3p partially reversed the inhibitory effect (Figure S3). After transfection of U87 and U251 cells with miR-339-3p mimics, miR-339-3p inhibitor, and LINC00467 overexpression plasmids, we tested the expression of IP6K2 by RT-PCR and Western blotting. The results suggested that miR-339-3p could inhibit the expression of IP6K2, whereas LINC00467 overexpression could partially reverse the inhibitory effect of miR-339-3p on IP6K2 (Figure 5(a-b)). Further, inhibiting miR-339-3p in glioma cells significantly promoted the expression of IP6K2 (Figure 5(a-b)). We then conducted EdU and Transwell experiments to measure the effect of the LINC00467/miR-339-3p/IP6K2 regulatory axis on the proliferation, migration, and invasion of glioma cells. The results indicated that after overexpression of miR-339-3p in U87 and U251 cells, the cell proliferation, migration, and invasion abilities were significantly reduced, whereas simultaneous LINC00467 overexpression partially reversed the inhibitory effect (Figure 5(c-e)). Further, transfection of cells with the miR-339-3p inhibitor promoted cell proliferation, migration, and invasion (Figure 5(c-e)). The above results thus demonstrated that the LINC00467/miR-339-3p/IP6K2 regulatory axis might promote the proliferation, migration, and invasion of glioma cells.

## Discussion

Glioma is the most frequent primary central nervous system tumor among neurosurgical diseases [31]. Early-stage patients may have no clinical symptoms. However, in patients with high-grade gliomas show a high degree of malignancy, rapid progression, high recurrence rate leading to short survival after diagnosis, and high fatality rates, which are highly challenging to treat [32,33]. Hence, it is urgent to explore novel biomarkers and potential mechanism of glioma.

LncRNAs can regulate gene expression both during transcription and post-transcriptionally. They can also regulate gene expression at the epigenetic level, and participate in various regulatory processes to perform different functions, including activation of transcription, interference with transfection, and nuclear transportation [34,35]. Abnormal expression may cause tumor cell proliferation or metastasis [36,37]. Previous studies have shown that lncRNAs play a vital role in the occurrence and progression of gliomas. For example, lncRNA LINC00998 can suppress the malignant glioma phenotype via the chromobox 3 (CBX3)-mediated c-Met/Akt/mTOR axis [38]. LncRNA brain cytoplasmic RNA 1 (BCYRN1) can suppress glioma tumorigenesis by binding with miR-619-5p to adjust CUE domain containing 2 (CUEDC2) expression and the PTEN/ AKT/p21 pathway [39]. Further, Linc-RA1 represses autophagy and improves radioresistance by avoiding H2Bub1/ ubiquitin specific peptidase 44 (USP44) combination in glioma cells [40].

In previous studies, we discovered that LINC00467 is significantly upregulated in glioma tissues, and the results of our in vitro cell experiments proved that LINC00467 could significantly facilitate glioma cell proliferation, migration, and invasion. We also found that LINC00467 is distributed in the nucleus and cytoplasm. Through bioinformatics analysis and cell experiments, we confirmed that LINC00467 in the nucleus could inhibit p53 expression by combining with DNMT1 to improve the malignant progression of glioma. However, the molecular mechanism of LINC00467 in the cytoplasm remains unknown.

In this study, we used bioinformatics analysis and discovered that IP6K2 is highly expressed in glioma tissues. Furthermore, we used RT-PCR to determine the expression level of IP6K2 in 30 glioma tissue samples and normal control tissues. The results showed that IP6K2 expression was significantly increased in glioma tissues. Moreover, the expression level of IP6K2 was correlated with the clinical features of glioma patients. In vivo and in vitro cell experiments showed that overexpression of IP6K2 could significantly promote the growth and metastasis of gliomas, indicating that IP6K2 might be involved in the malignant progression of gliomas as an oncogene.

The biological function of lncRNA is related to its subcellular location. LncRNAs can absorb miRNAs by acting as ceRNAs, thereby inhibiting mRNA expression. This type of regulation occurs mainly in the cytoplasm. With the understanding of the regulatory mechanism of ceRNA, many researchers have turned their attention to ceRNA, trying to explore its regulatory role in tumors and other diseases, in order to enrich the understanding of tumor pathogenesis. For example, Lnc-DLEU2 can drive epithelial-mesenchymal transition and glycolysis in endometrial cancer through HK2 by competitively binding to miR-455 and by modulating the enhancer of zeste 2 polycomb repressive complex 2 subunit (EZH2)/miR-181 pathway [41]. Lnc- AFAP1 antisense RNA 1 (AFAP1-AS1) can promote tumor progression and invasion by regulating the miR-2110/Sp1 axis in triple-negative breast cancer [42]. LINC00467 is also distributed in the cytoplasm of glioma cells, suggesting that LINC00467 may play an important supervisory role at the post-transcriptional level. We thus investigated whether LINC00467 could regulate the expression of IP6K2 as a ceRNA in the cytoplasm. Bioinformatics analysis indicated miRNAs that can simultaneously bind LINC00467 and IP6K2, among which miR-339-3p showed the strongest binding ability. MiR-339-3p has been proven to exert tumor suppressor effects in colorectal cancer [43], melanoma [44] and other tumors. The dual luciferase reporter gene assay confirmed that miR-339-3p could simultaneously bind to LINC00467 and IP6K2. Further testing revealed that LINC00467 could bind to miR-339-3p and promote the expression of IP6K2. This indicates that LINC00467 might play a role in promoting cancer by binding to miR-339-3p.

**Figure 6.**
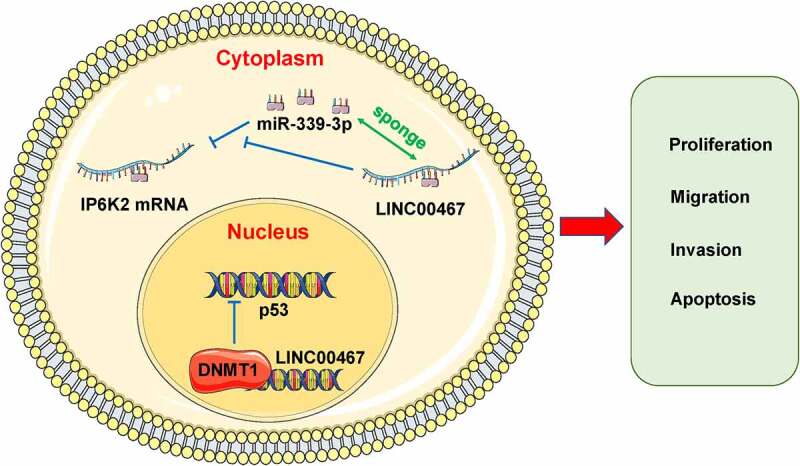
The potential molecular mechanism of LINC00467 in glioma.

IP6K2 is a tumor-promoting gene that is highly expressed in gliomas, but its possible molecular mechanism is unknown. Our previous study revealed that LINC00467 is distributed in the nucleus and cytoplasm of glioma cells. In previous studies, we initially explored the possible molecular mechanism of LINC00467 in the nucleus. In this study, through a range of in vitro experiments and bioinformatics analyses, we preliminarily proved that LINC00467 might promote IP6K2 expression by binding with miR-339-3p to promote the proliferation, migration, and invasion of gliomas. In general, we propose that LINC00467 could inhibit p53 expression in the nucleus by binding to DNMT1, while it might promote IP6K2 expression by binding to miR-339-3p in the cytoplasm, thereby promoting the malignant growth and metastasis of gliomas (Figure 6).

However, this study still has several shortcomings. First, this study did not use fluorescence in situ hybridization (FISH) to conduct an in-depth study regarding the subcellular localization of LINC00467. In addition, rescue experiments could further prove the role of IP6K2 and determined whether the inhibitor/activator of the DNMT1/p53 signaling pathway can reverse the cellular function of LINC00467. Moreover, the number of clinical cases collected in this study was relatively small, and although the statistical results obtained were relatively reliable, more clinical tissues could be collected in the future to determine the expression of LINC00467. Furthermore, clinical follow-up results should be combined to identify the clinical application value of LINC00467 in glioma.

## Conclusion

This study preliminarily proved that LINC00467 might boost the proliferation, migration, and invasion of glioma cells by binding with miR-339-3p to promote the expression of IP6K2. The LINC00467/miR-339-3p/IP6K2 regulatory axis might thus be a biomarker and potential therapeutic target in glioma.

## Supplementary Material

Supplemental Material
