# Supplementary figures and images for: LINC00467 facilitates the proliferation, migration and invasion of glioma via promoting the expression of inositol hexakisphosphate kinase 2 by binding to miR-339-3p
Source: Bioengineered. 2022 Feb 13;13(2):3370–82. doi: 10.1080/21655979.2021.2018098 (PMC8973818; doi:10.1080/21655979.2021.2018098)

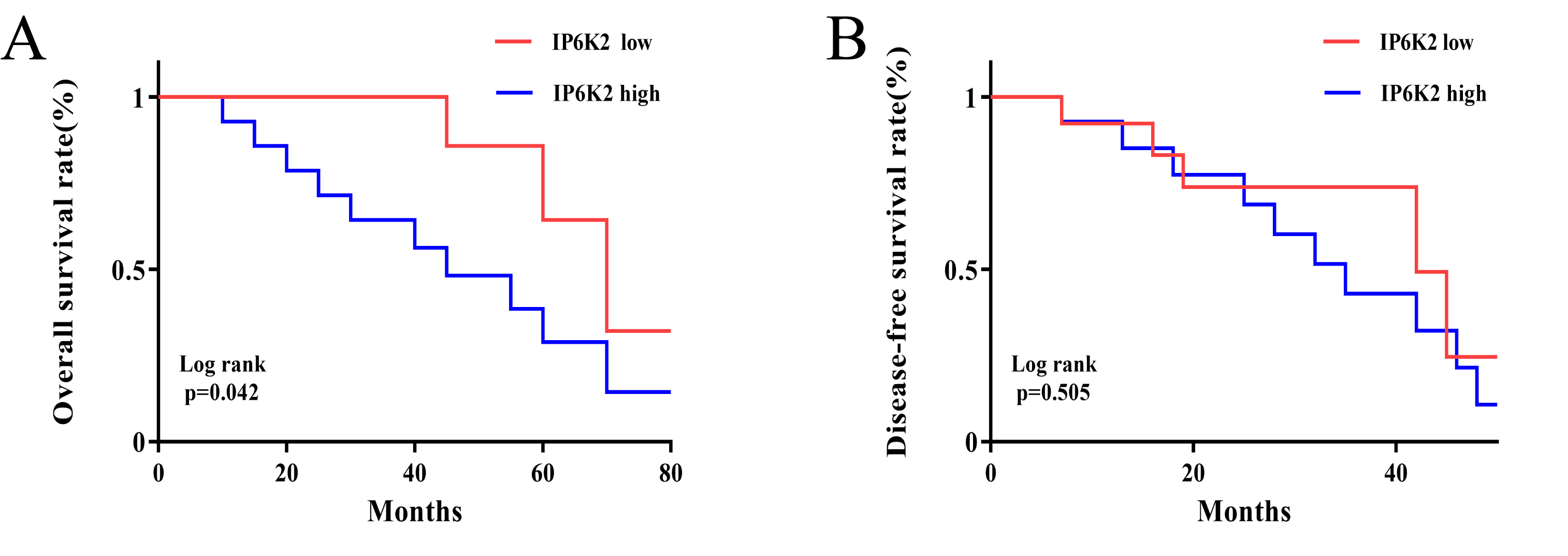

Supplement: Supplemental Material [file KBIE_A_2018098_SM3499.zip › supplementary/Figure S1.tif]

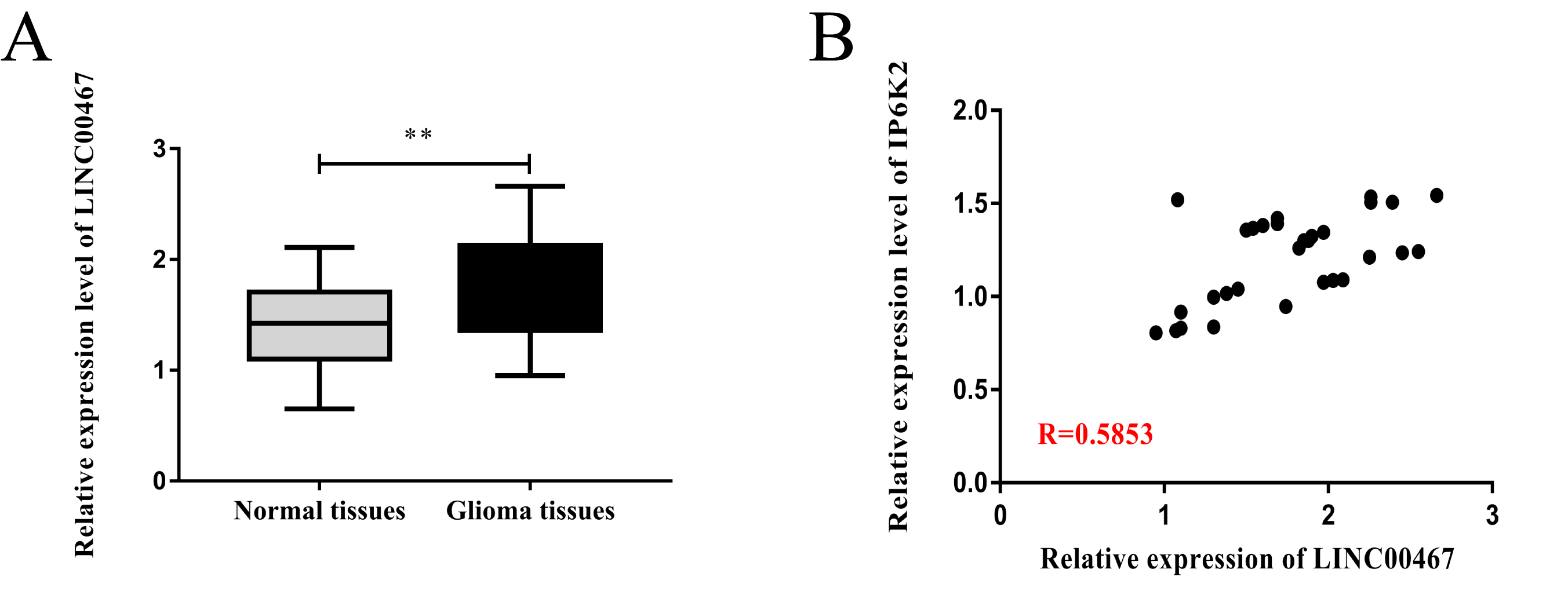

Supplement: Supplemental Material [file KBIE_A_2018098_SM3499.zip › supplementary/Figure S2.tif]

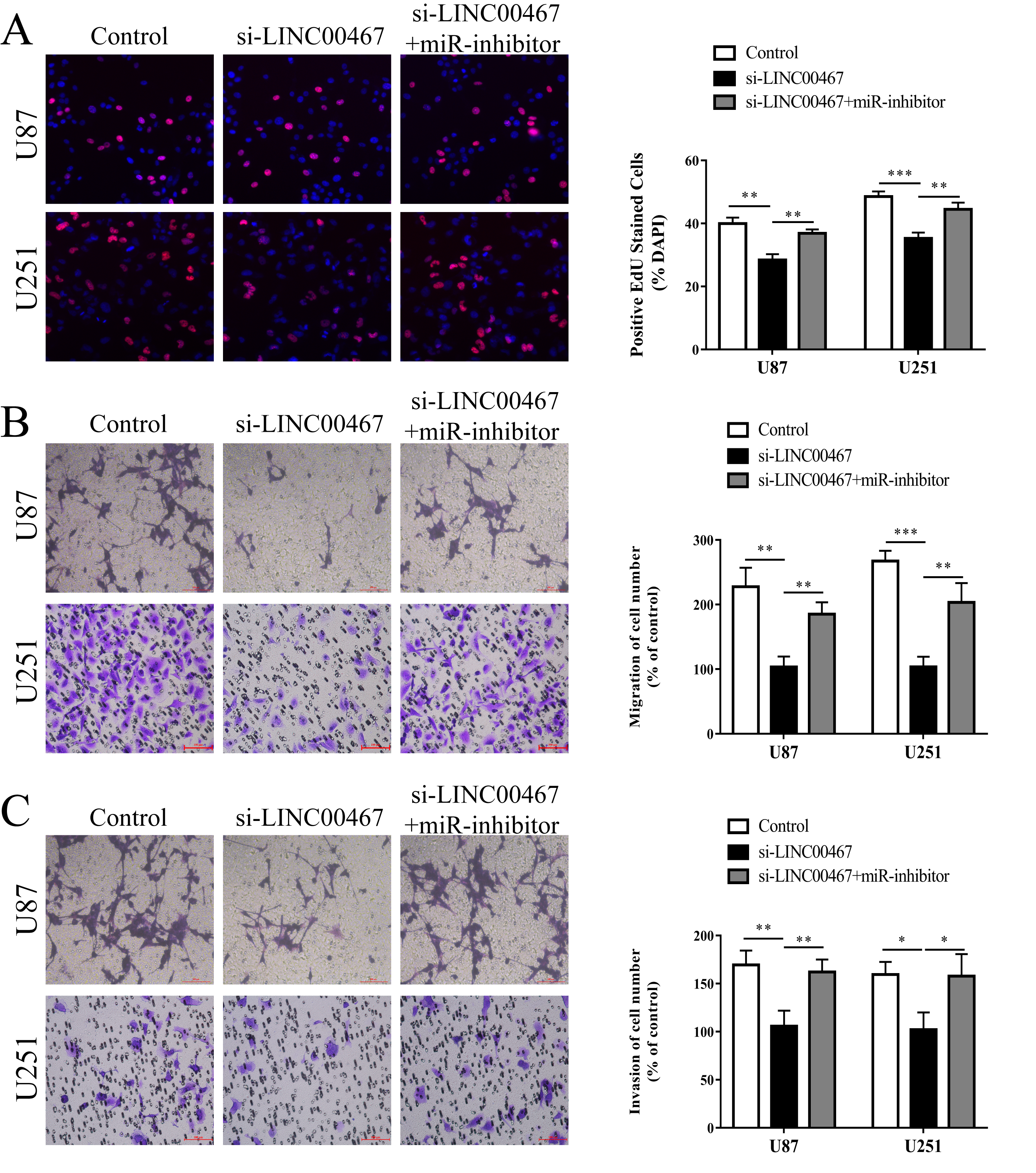

Supplement: Supplemental Material [file KBIE_A_2018098_SM3499.zip › supplementary/Figure S3.tif]
